# Editorial: Oxytocin's routes in social behavior: into the 21st century

**DOI:** 10.3389/fnbeh.2015.00224

**Published:** 2015-08-25

**Authors:** Elissar Andari

**Affiliations:** Center for Translational Social Neuroscience, Silvio O. Conte Center for Oxytocin and Social Cognition, Division of Behavioral Neuroscience and Psychiatric Disorders, Yerkes National Primate Research Center, Emory UniversityAtlanta, GA, USA

**Keywords:** oxytocin, precision medicine, adaptive behavior, social behavior, psychiatric disorders

The role of the neuropeptide oxytocin in social behaviors is one of the earliest discoveries in social neuroscience. Influential studies in animal models have delineated many of the neural circuits and genetic components that underlie these behaviors (Insel and Young, 2001; Donaldson and Young, 2008). These discoveries have inspired researchers to investigate the effects of oxytocin on brain and behavior in humans (Kirsch et al., 2005; Kosfeld et al., 2005) and its potential as a treatment for psychiatric disorders (Hollander et al., 2007; Andari et al., 2010; Guastella et al., 2010).

Several criticisms have been raised lately on the effects of intranasal oxytocin (IN-OT) in humans such as weak effect sizes, lack of dose studies and ambiguity about chronic administration. We clearly need more empirical data before drawing conclusions on IN-OT. Yet, how do we approach these needs and how are we making progress with IN-OT or other oxytocin-targeted drugs? Increasing the sample size and performing replications are important factors (Walum et al., 2015), but are only part of the answer. We cannot minimize the importance of innovative medium-sized studies that are based on theoretical frameworks or constructive hypotheses (or even chance observations) that form the foundation of seminal discoveries. Even single-case studies have advanced the field of neuroscience dramatically. Combining large data sets without a theory-based approach and meticulous view of behavior and symptoms can lead to a dilution of important findings and to misleading conclusions. Recent meta-analyses of the effects of IN-OT across all psychiatric disorders and healthy subjects show that the overall effect sizes are low (Bakermans-Kranenburg and Van, 2013; Walum et al., 2015), which some have interpreted to mean that all the effects of IN-OT in humans are likely to be false positives and not true effects (Walum et al., 2015). However, the effects on specific disorders (such as Autism Spectrum or ASD) are medium to high, which indicates that IN-OT can be more beneficial for ASD than for psychiatric disorders in general (Bakermans-Kranenburg and Van, 2013; Young and Barrett, 2015). Oxytocin's role in ASD is further supported by a seminal study showing that chronic IN-OT early in life restored later social behavior in a mouse model of autism (Penagarikano et al., 2015). Other disorders, such as PTSD (Post Traumatic Stress Disorder), might need further investigation. It has been recently observed that IN-OT facilitates extinction of conditioned fear in healthy subjects (Eckstein et al., 2015). Also, IN-OT is more beneficial for some individuals than others within healthy and ASD populations (Andari et al., 2010; Bartz et al., 2010; Marsh et al., 2012; Riem et al., 2014) and some studies include hypotheses and outcome measures that represent oxytocin's effects and its function better than others.

*More research on how individual social aptitudes and emotional regulation, clinical characteristics, receptor distribution and genetic polymorphisms can affect the social outcome of oxytocin-based treatments is needed*. It is crucial to know more precisely who can benefit from oxytocin-related treatments, which outcome measures will best represent its effects, how it should be administered, and what are the brain mechanisms. This “precision medicine” approach for IN-OT is in line with the Research Domain Criteria of the U.S. National Institute of Mental Health (Cuthbert and Insel, 2013).

This special issue collects 12 interesting papers on the many facets of the oxytocin system and social behavior. This topic embraces a very interesting aspect of oxytocin biology, which is the evolution of its function (Knobloch and Grinevich, 2014, this issue). Oxytocin-like genes and peptides are highly conserved and homologs are even observed in primitive invertebrate species such as insects and mollusks. However, the oxytocin system went through tremendous microanatomical and cytological transformations during evolution. Along with the development of the forebrain, the ancestral preoptic nucleus in basal vertebrates (fish) diverged into a polycentric oxytocin system (paraventricular and supraoptic nuclei) in advanced vertebrates (reptiles, birds and mammals). Oxytocin neurons acquired a voluminous dendritic trees and branching axons along with an expansion of oxytocin axonal projections to forebrain regions, *a physiological advancement that shapes the complexity of evolved social behaviors*. This topic also includes a novel finding on the distinct regional developmental changes in oxytocin receptor density (OXTR) over postnatal time in a mouse model (Hammock and Levitt, 2013, this issue). OXTR density or distribution or gene polymorphisms can shape social behavior and can be a moderator of the degree of susceptibility to early adverse experiences (Anacker and Beery, 2013, this issue; Parker et al., 2014; Barrett et al., 2015). In this issue are two genetic imaging papers that showed correlations between polymorphisms of the OXTR gene and social outcome and the BOLD activity of social brain regions in humans (Verbeke et al., 2013, this issue; Michalska et al., 2014, this issue). Not only does oxytocin system shape social outcomes, environmental factors like early-life stress can also impact the oxytocin system such as its endogenous release (Crockford et al., 2014, this issue).

Also included in this topic are two papers on rhesus macaques, which are a valuable model for examining the neurobiology of socioemotional behaviors due to the physiological and social complexity that they share with humans. Authors found that peripheral measures of oxytocin and vasopressin in juveniles rhesus monkeys are influenced by early involvement in friendship (Weinstein et al., 2014, this issue). In another interesting study, IN-OT enhanced socially-reinforced learning in rhesus monkeys which were trained on an implicit visual matching task (Parr, 2014, this issue). These results are, in part, congruent with the model proposed in this special issue stating that IN-OT is likely to increase reward sensitivity and reduce anxiety in order to enhance social salience and social cognition (Bethlehem et al., 2014, this issue).

There is a current debate in the literature on whether oxytocin is promoting positive prosocial behaviors or whether it is triggering negative behaviors such as defensive mechanisms. In this special issue, authors found that IN-OT increased the bias for “liking” in-group stimuli and did not increase out-group derogation in humans (Ma et al., 2014, this issue). IN-OT is not necessarily the peace or the war molecule; its role in social functioning is likely to be more complex*. Its action is likely to be adaptive in impacting a variety of socio-emotional behaviors based on contextual demands and individual predispositions*, possibly through its interaction with GABAergic neurons (Tyzio et al., 2014), serotonin (Mottolese et al., 2014), reward system (Burkett and Young, 2012) and neuronal signaling (Owen et al., 2013; Marlin et al., 2015). Pro-social behaviors but also dominance, defensive behaviors and other behaviors are necessary for our social structure and adaptation. In a very interesting review (Ebitz and Platt, 2013, this issue), authors stressed how for a greater evolutionary fitness, oxytocin's function is adaptive and is species-specific. Notably, in another review, authors discussed the variability and the different types of social behavior (aggression, engagement, grooming, dominance) between species (Anacker and Beery, 2013, this issue), a fact that should be taken into account when drawing conclusions on the effects of oxytocin on behavior.

Despite the progress in this field, there are important questions that still need to be addressed. Who can benefit from oxytocin intake? How do we measure the predicted value of oxytocin-based benefits? Does IN-OT penetrate the brain? IN-OT increases oxytocin levels in the brain (Neumann et al., 2013; Striepens et al., 2013) and increases brain activity and resting cerebral blood flow (Domes et al., 2013; Gordon et al., 2013; Paloyelis et al., 2014). More research dissociating exogenous from endogenous effects of oxytocin can lead the field forward. Importantly, in this special issue, authors raised precautions about the risks and the effects of chronic IN-OT in pediatric populations (Taylor et al., 2014, this issue). A few chronic IN-OT studies have shown that it is safe in young (7–18 years old) and adult populations but with ambiguous social outcomes (Anagnostou et al., 2012; Tachibana et al., 2013; Dadds et al., 2014; Guastella et al., 2015). Recently, it has been shown that stimulating the oxytocin system endogenously with melanocortin-4 receptor agonist (Barrett et al., 2014; Modi et al., 2015) or MDMA (Dumont et al., 2009) is promising but also needs more investigation. More research on the primary outcome measures, the methods of treatment (drugs, doses, chronic administration with behavioral therapies), and the individual variability in endogenous oxytocin release following new drug treatments and their side effects, is needed.

Oxytocin is an exciting target for improving social function, yet there is a gap between theory and actual therapeutic applications. Studying interactions between neuropeptides, brain mechanisms of social functions, inter-individual variability, and combining neurobiology with behavioral therapies can lead to better outcomes. *With a powerful sense of determination and optimism, respect of each other fields, multidisciplinary collaborations, careful replications and adoption of a “precision medicine” approach within fundamental science and human research; we together can help men and women who suffer from brain dysfunctions*.

## Funding

EA is supported by National Institute of Health (NIH) grant (1P50MH100023 entitled Silvio O. Conte Center for Oxytocin and Social Cognition).

### Conflict of interest statement

The author declares that the research was conducted in the absence of any commercial or financial relationships that could be construed as a potential conflict of interest.
